# Robotic assisted-bronchoscopy: technical tips and lessons learned from the initial experience with sampling peripheral lung lesions

**DOI:** 10.1186/s12890-019-0857-z

**Published:** 2019-05-09

**Authors:** Septimiu Dan Murgu

**Affiliations:** The University of Chicago Medicine, 5841 S. Maryland Avenue, Chicago, IL 60637 USA

**Keywords:** Peripheral lung nodule, Peripheral lung lesion, Robotic bronchoscopy, Navigation bronchoscopy, Radial probe ultrasound, Guided bronchoscopy, Lung cancer, Screening

## Abstract

**Background:**

Peripheral pulmonary nodules are increasingly detected in patients screened for lung cancer or during disease progression of thoracic or extrathoracic malignancies. Sampling these lesions requires surgery, computed tomography (CT)-guided biopsy or bronchoscopic interventions. Bronchoscopic interventions are preferable because they have lower complications and often patients may not be ideal candidates for surgical or CT-guided biopsy. In addition, guidelines recommend diagnosis and staging in one single procedure. The diagnostic yield of existing advanced bronchoscopic techniques including electromagnetic navigation, radial probe ultrasonography, ultrathin bronchoscopy or virtual bronchoscopy remains suboptimal. The purpose of this paper is to codify the technique whereby a diagnostic bronchoscopy is performed using the new robotic platform.

**Methods:**

In the present report, I describe the technique for performing robotic-assisted bronchoscopy (RAB) using the Monarch™ platform (Auris Health, Inc., Redwood City, CA).

**Results:**

Appropriate team training, patient selection, anesthesia settings, optimal tissue acquisition and processing, and prevention of complications are described and illustrated.

**Conclusions:**

RAB may be beneficial for patients with peripheral lung lesions that require biopsy prior to surgical resection, stereotactic radiation, targeted or immunotherapy.

## Background

Detection of lung nodules in screening trials resulted in a new challenge for clinicians involved in caring for these patients. The National Lung Screening and NELSON trials showed that a significant number of patients had a positive screen [1, 2]. The vast majority (~ 80%) of the screen-detected nodules are located in the periphery of the lung. The American College of Chest Physician and National Comprehensive Cancer Network guidelines recommend obtaining the diagnosis of the primary lesion, staging, genomic alterations and PD-L1 status in the least invasive manner and ideally in a single procedure [3, 4]. In addition, guidelines recommend re-biopsy at the time of disease progression, as genomic alterations could result in change of therapy in certain patients or could allow for enrollment in clinical trials [4]. In these regards, based on the size or location of the nodule, the finding of fluorodeoxyglucose-avid intrathoracic adenopathy on positron emission tomography or enlarged nodes on computed tomography (CT), concurrent mediastinal staging with endobronchial ultrasound-transbronchial needle aspiration is indicated [5]. Thus, in these patients, an initial bronchoscopic approach for staging and biopsy of the lesion is warranted. The increasing need to efficiently and safely sample lung lesions has led to the development of virtual bronchoscopy (VB), radial endobronchial ultrasound (r-EBUS), electromagnetic navigation (EMN) and ultrathin bronchoscopes. The diagnostic yield using these bronchoscopic techniques remains suboptimal [6, 7]. The now commercially available robotic bronchoscopy platform (The Monarch™ platform, Auris Health, Inc., Redwood City, CA) has the potential to overcome some of these limitations. A small feasibility study that enrolled 15 patients using the RAB was performed and showed no pneumothoraces or significant bleeding [8]. In cadaveric models, RAB was shown to have farther access to the periphery of the lung when compared to 4.2 mm OD conventional thin bronchoscopes (9 vs 6 airway generations) [9].

The purpose of this paper is to describe the technique of RAB performed with the Monarch platform.

## Methods

The relationship between human airway anatomy and components of the platform is described. The basis for this description is the experience in performing conventional bronchoscopies as well as robot-assisted bronchoscopy with the novel platform. Due to recent increased interest in robot-assisted technologies, it is important to describe the technique that maximizes incorporated technological advancements with an emphasis on optimizing yield and safety. Suggestions are given around appropriate team training, patient selection, anesthesia settings, optimal tissue acquisition and processing, and prevention of complications. Specifically, the paper: 1) describes how dedicated team training can significantly decrease system and room set up time; 2) illustrates the pros and cons of mental and computer-based navigation planning; 3) shows how to optimize farther advancement of the scope by adjusting positive end-expiratory pressure (PEEP) level and using forceps for modified Seldinger technique; 4) explains how to optimize bronchoscopic visualization once in the distal airways by allowing pressure equilibration between the atmosphere and the target airway; 5) demonstrates how to identify the exact location of the lesion by visualizing the contact between the r-EBUS probe and airway wall; 6) reveals how to optimize specimen processing by avoiding saline and by using air insufflation during scope advancement; and 7) describes the tools and their application sequence for sampling peripheral lung lesions.

## Results

### Robotic Bronchoscopic system

The main components of the novel robotic bronchoscopy platform include the Bronchoscope System, Cart and Tower. Several external components such as fluidics control, electro-magnetic (EM) field generator and reference EM sensors interface with the Cart. The current platform received the United States Food and Drug Administration clearance in March 2018.

**Fig. 1:**
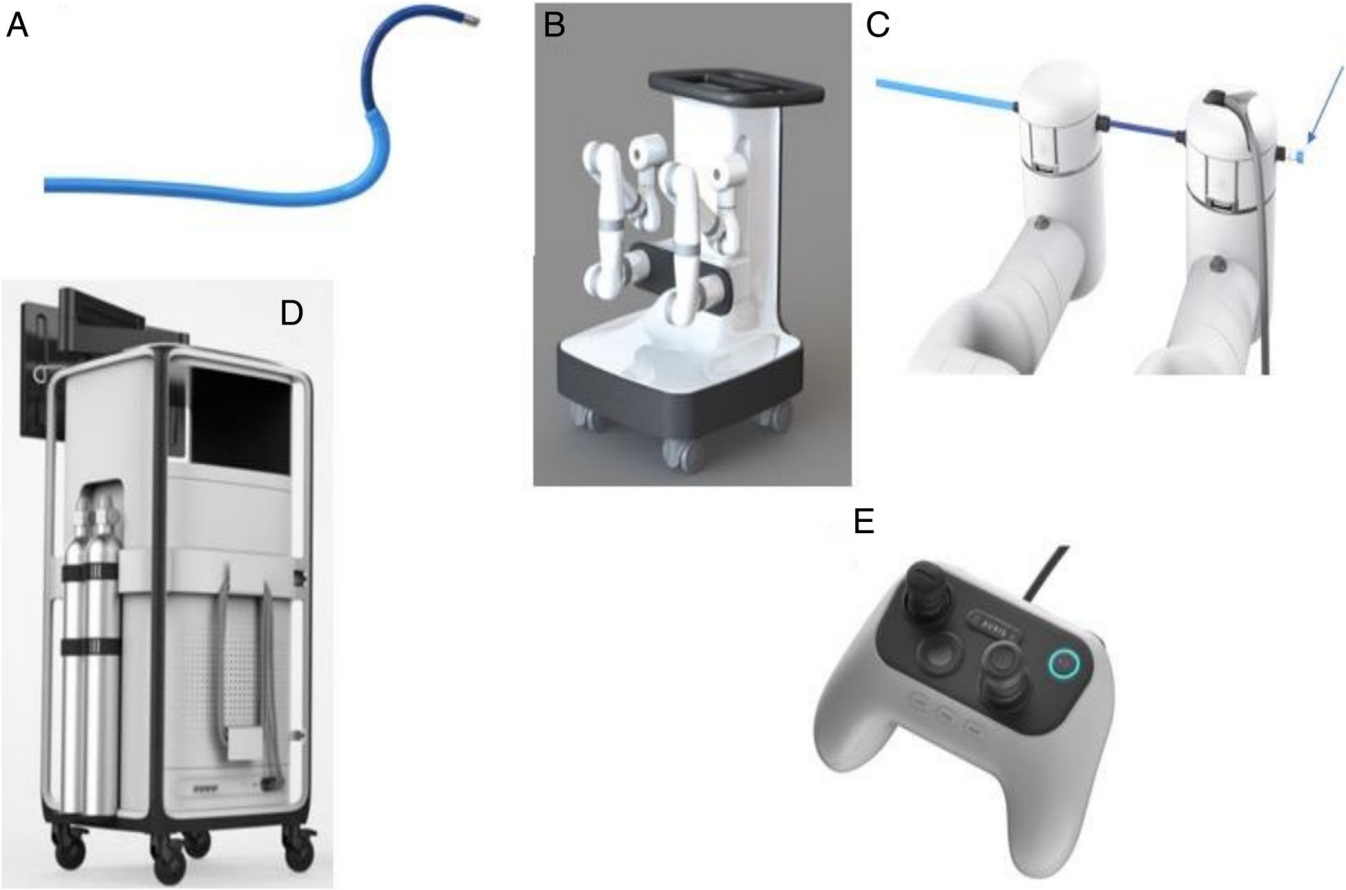
**a** The Auris Bronchoscope is formed by an inner scope and the outer sheath. The Bronchoscope includes a camera that provides the operative perspective, an integrated light source in the handle and a 2.1 mm inner diameter working channel for the passing of manually controlled tools. **b** The Auris Cart with the robotic arms. **c** Attachment of the bronchoscope to the robotic arms with the proximal valve for saline, air or instrument insertion (arrow). **d** The Tower with the monitor for endoscopic and electromagnetic navigation display. **e** The Controller. Photos courtesy of Auris Health, Inc., Redwood City, CA

The Bronchoscope System is formed by an inner bronchoscope and the outer sheath, both of which possess 4-way steering control (Fig. 1a). This configuration enables the capability of telescoping, which enhances the bronchoscope stability and access capability further into the lung. The bronchoscope includes a camera that provides the operative perspective, an integrated light source in the scope handle and a 2.1 mm inner diameter working channel for the passing of tools. The bronchoscope and sheath have a distal section capable of achieving articulation in pitch, yaw and any combination of the two to enable precise control while driving the bronchoscope (Fig. 1a). Proximally, the bronchoscope is equipped with a valve to facilitate the insertion and sealing of various ancillary devices, such as needles or biopsy forceps. Additionally, the proximal section routes irrigation and aspiration to the shared working channel.

The Cart includes two robot arms which contain rotary pulleys to actuate the drive cables in the bronchoscope (Fig. 1b). The Cart houses the electronic systems required to power and operate the platform. Automated lift controls will raise and lower the height of the robotic arms, which connect to the bronchoscope system (Fig. 1c). The cart handle allows the cart to be maneuvered so that the cart wheels can be directionally locked. An embedded touchscreen on the cart handle provides feedback during system setup.

The Tower (Fig. 1d) provides connectivity for the bronchoscope camera and lighting, as well as the fluidics system and includes a controller that allows the clinician to control the system during the procedure (Fig. 1e). On the controller, two joysticks are used to drive and articulate the bronchoscope while various buttons are used to control irrigation, aspiration and the device state (Fig. 1e). The Tower houses two computers that run the system, a Non-Real Time Computer and a Real-Time Computer. The Non-Real-Time computer takes the inputs from the pendant, keyboard, mouse, camera, EM Localization/Targeting System and Power Distribution Unit. The Non-Real-Time computer also contains an interface to the micro camera at the tip of the bronchoscope. The camera interface performs the necessary image processing and generates output video streams. The robotic system algorithms are also implemented on the Real-Time computer. The Real-Time computer receives inputs from the Non-Real-Time computer. The network handles communication between the two computers, robotic arms and Power Distribution Unit. A single monitor is integrated into the Tower to display real time video captured from the bronchoscope camera overlaid with information on the status of the robotic system.

The system has several components that interface to the Auris Cart including: Fluidics Control, EM Field generator, and Reference EM sensors. The Fluidics Control consists of a peristaltic pump and controlled valves. The fluidics control can dispense a fluid through a single-use tubing set into the endoscope and actuates aspiration of fluids to an external vacuum source. The EM Field generator is used as part of the system for navigation guidance. The Reference EM sensors are used to monitor the patient position relative to the EM Field Generator.

### Technique

#### System set up

**Fig. 2:**
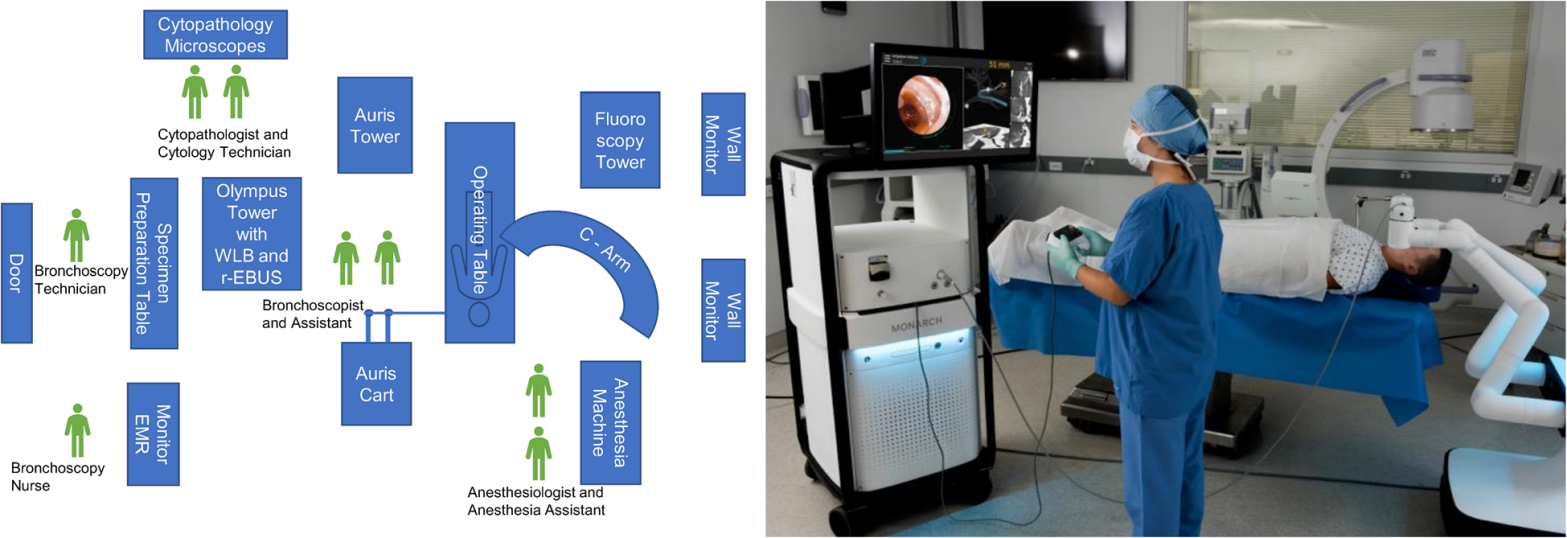
Schematic representation of the room set up for robotic assisted bronchoscopy (left panel). Real life picture of the Auris tower, cart, operating table and fluoroscopy system (right panel- photo courtesy of Auris Health, Inc., Redwood City, CA.) REBUS: radial probe ultrasound; EMR: electronic medical records; WLB: white light bronchoscopy

Dedicated team training can significantly decrease system and room set up time. Becoming familiar with the Bronchoscope System, Cart and Tower requires proper training and a relatively stable operating team. Our team at the University of Chicago (U of C) had been in-serviced on two occasions prior to implementing RAB in clinical practice. On-site technical support from the robot manufacturer is offered on a regular basis. At the time of this writing, after more than 50 cases performed at our institution, the system set up lasts less than 10 min. At the U of C, the room arrangement is as illustrated in Fig. 2. It is also important to remember to remove large metallic objects from the operating table and surrounding the EM field generator during the set up and EM navigation phase of the procedure. These include but are not limited to the metallic anesthesia tubing support system, metallic stepping stools and fluoroscopy system.

#### Anesthesia considerations

At the U of C, we use general anesthesia with an indwelling endotracheal tube for all RAB procedures. Neuromuscular blockade is used at a discretion of the anesthesia team. The goal is to maintain a quiet field for the time of the procedure. Airway inspection using a conventional white light bronchoscope is performed prior to RAB to rule out an obvious endobronchial lesion and to suction secretions from the airways. When mediastinal staging is indicated, EBUS-guided transbronchial needle aspiration is performed prior to RAB. During RAB, a tidal volume of ~ 8 ml/kg ideal body weight and a PEEP of 8–10 cm H_2_O is provided with the attempt to splint open the small, subsegmental airways. Application of PEEP or even a brief recruitment maneuver facilitates advancement of the scope beyond 5th–6th generation without the need to inject saline. This may be relevant to those operators who plan to perform r- EBUS or cone beam CT imaging, as saline in the alveolar space around the target lesion will lead to alveolar filling and possibly false positive r- EBUS images. In addition, for those who use rapid on-site cytological evaluation (ROSE), saline on the cytology slide may disrupt the cells and negatively effect on site specimen interpretation.

#### Navigation

**Fig. 3:**
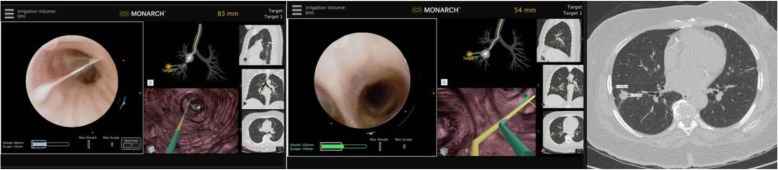
Manually (in yellow) and automatically (in green) generated pathways to a peripheral nodule in RB8 (anterior basal segment of the right lower lobe). The two pathways overlap in the large central airways (left panel) but clearly diverge in 5th generation airway (middle panel). The bronchoscopist must decide which pathway to follow first. In our practice, we follow the manually generated pathway and confirm it with REBUS once at target. If the nodule is not seen on REBUS, the scope is pulled back and the automatically generated pathway is then followed. The right panel shows the nodule biopsied in this case (a 15.8 X 15.7 mm nodule in RB8) REBUS: radial probe ultrasound

**Fig. 4:**
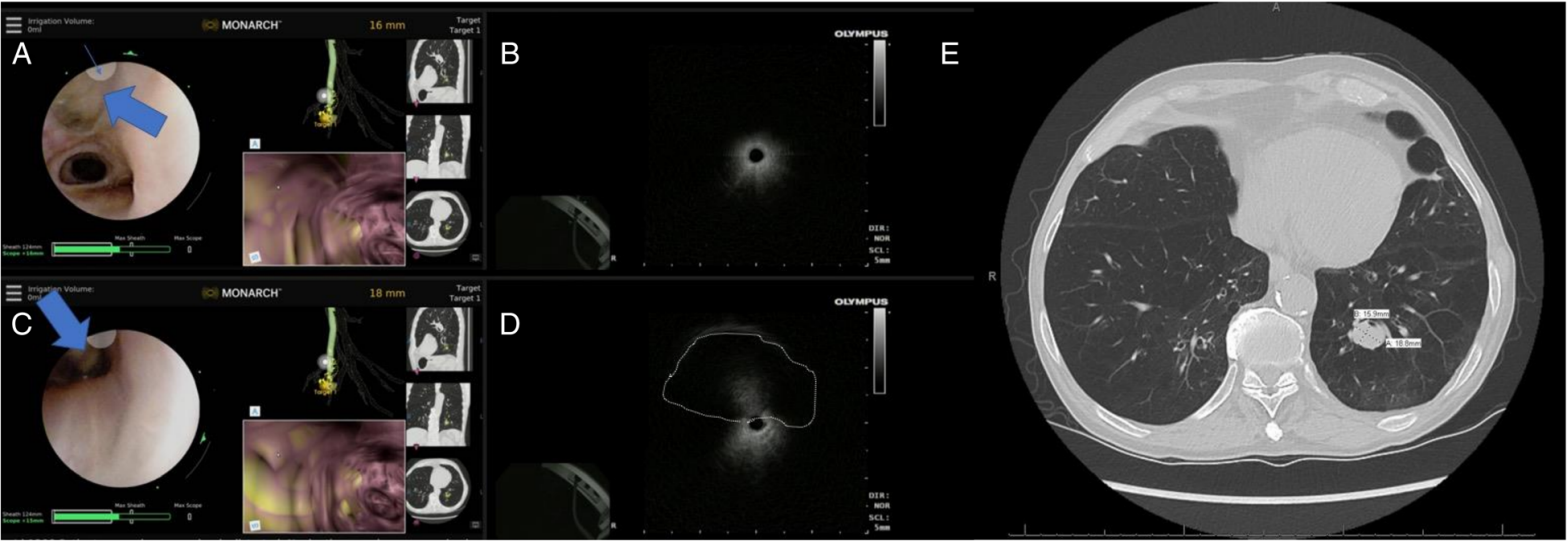
REBUS- nodule relationship during robotic bronchoscopy. **a** REBUS probe (thick arrow) is advanced under direct visualization and seen exiting the scope at the site of the indicator (at 11 o’clock position on the screen- thin arrow). **b** in that airway, the REBUS image only showed air artifact. **c** In the same location, the REBUS probe is then oriented towards 6 O’clock position on the screen. The probe is in partial contact with the airway wall (thick arrow). **d** In that position, REBUS screen shows the nodule as an isoechoic image- highlighted with a dashed line). It is also obvious that nodule as seen on REBUS, although it appears to be at 12 o’clock position relative to the probe, it is clearly at 6’o’clock position as that is where the probe touches the airway wall. This relationship is crucial to identify as the needle orientation is dependent on it. **e** The panel shows the chest CT scan with the nodule biopsied in this case (a 15.9 X 18.8 mm nodule in RB10) REBUS: radial probe ultrasound

**Fig. 5:**
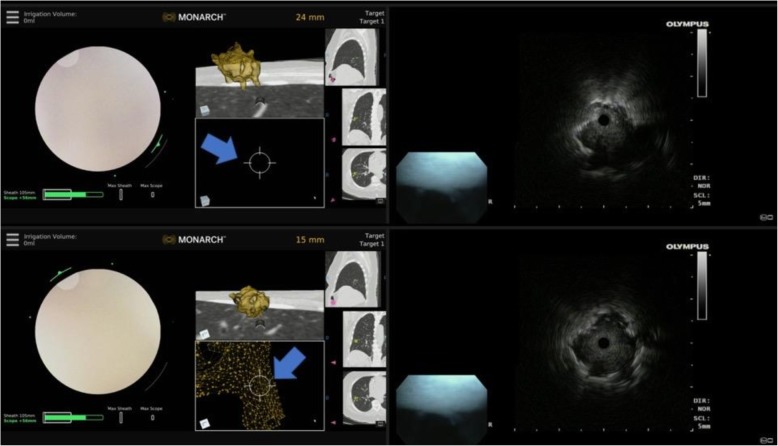
Eccentric and concentric REBUS image patterns during robotic bronchoscopy. Top panel: at 24 mm away from the nodule, the REBUS probe was advanced and only an eccentric pattern was obtained; note that the target is not seen on the Monarch EMN display monitor (blue arrow). Bottom panel: the scope is advanced to 15 mm away from the lesion, and the REBUS monitor now shows a concentric pattern (associated with higher diagnostic yield). Note that the nodule is now also seen on the Monarch EMN display monitor (blue arrow). REBUS: radial probe ultrasound; EMN: electromagnetic navigation

A controller is used to move the robotic arms that contain rotatory pulleys to drive the bronchoscope. The outer sheath of the robotic bronchoscope system is 6.0 mm in diameter and could be wedged in a segmental or even subsegmental airways. By doing this, the rest of the target lobe, ipsilateral and contralateral lung are protected in case of biopsy-related bleeding. However, wedging the sheath too far (beyond 4th generation airway) may impede the manipulation of the inner scope as it comes out of the sheath. This is because the maneuvering of the inner scope in the small and short branching airways could be difficult if the outer sheath is wedged to far. In this case, the outer sheath is slightly retracted to segmental or lobar airways. The inner bronchoscope with an OD of 4.2 mm is then advanced to the target lesion by following the manually or automatically generated pathways (Fig. 3). The EMN system uses an EM field generator and reference sensors much like other EMN bronchoscopy systems. In patients with defibrillators and pacemakers, the EMN system is currently not recommended by the manufacturer. In these patients, we chose not to use the EMN but just the robotic bronchoscopic system for the purpose of stability and farther reach in the lung periphery. Prior to each case, in fact, we create our own path not only on the system’s software, but also by independent review of the CT scan and by identifying the airways leading to or in the proximity of the target lesion. We have diagnosed lesions with and without the classic “bronchus sign” as long as a blood vessel is noted adjacent or leading to the lesion. Based on knowledge of the pulmonary lobule anatomy and on extensive prior experience with EMN bronchoscopy, lesions without a “bronchus sign” but with a blood vessel leading to them, do have an adjoining airway even if not obvious on the planning chest CT scan. If a small airway (usually beyond 5th generation) is difficult to access, we learned to use biopsy forceps or the r-EBUS probe for a modified Seldinger technique to allow sliding the scope over the forceps/ probe. Once the target is reached based on the EMN system feedback, we routinely perform radial probe ultrasonography to confirm correct navigation and identify the actual relationship between the airway and the lesion (Figs. 4 and 5). Once this relationship is identified, the sampling tools are inserted and specimen is collected. Sampling tools are advanced through the working channel (2.1 mm), as is done in conventional flexible bronchoscopy, to sample the lesion under fluoroscopy.

#### Workflow at target

**Fig. 6:**
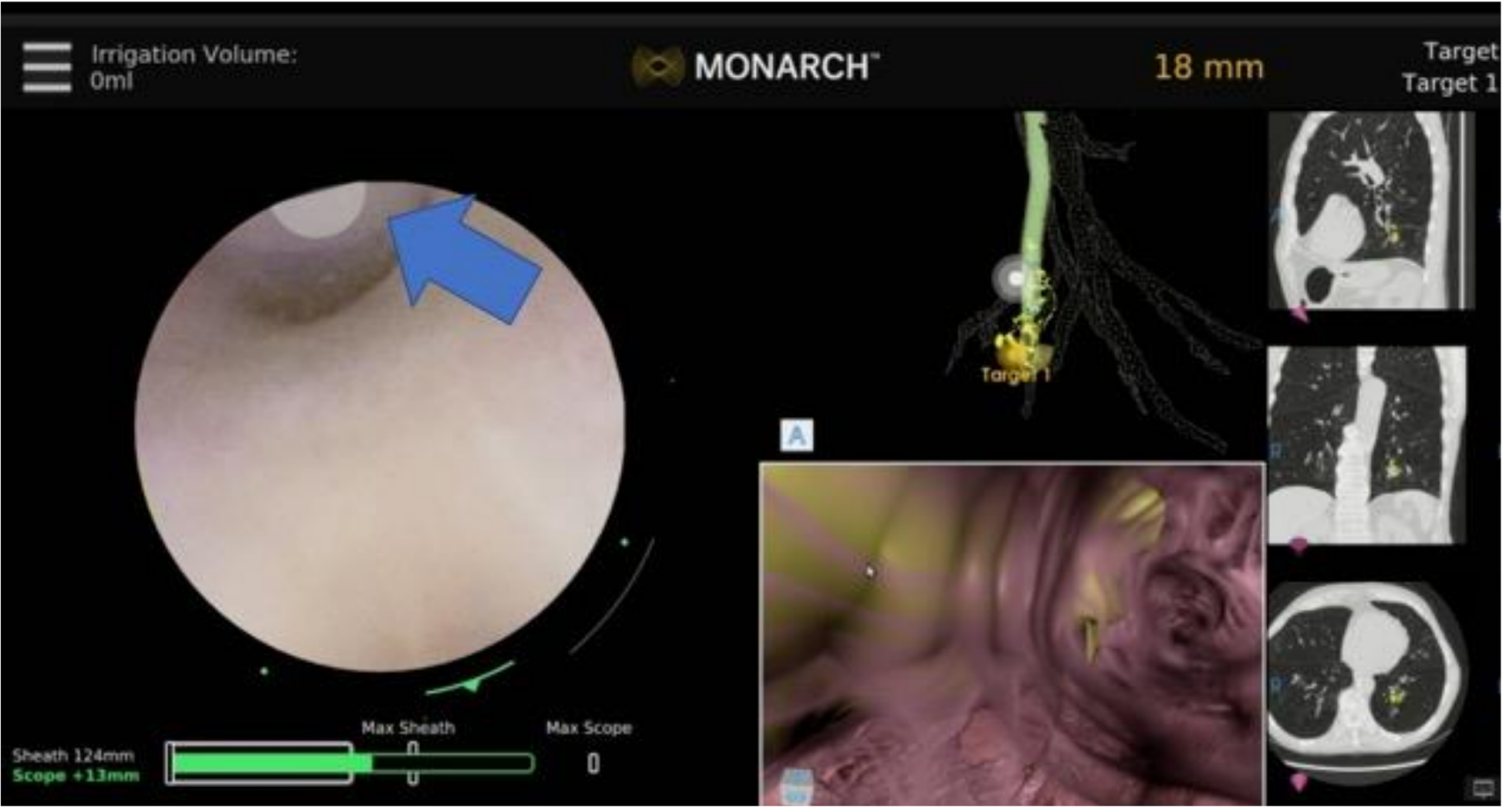
Needle aspiration during RAB. The Auris needle is seen exiting the bronchoscope at 11 o’clock position (thick arrow). The needle can be re-oriented as necessary, while the bronchoscope is locked in position, but the tip is free to rotate in the desired location. RAB: robotic-assisted bronchoscopy

We start by using the Auris needle (Fig. 6) but other needles (eg. 21 g Olympus Periview Flex TBNA, Olympus America) can be used with Auris bronchoscope. Three to four needle aspirations are performed under fluoroscopy and Diff Quick smears are prepared. If the diagnosis is made and no tissue is necessary for ancillary studies, the procedure could be stopped at that point. The direction of the needle can be reoriented under direct visual guidance (Fig. 6). In many cases, however, histology type of material is needed for a variety of ancillary tests. Or, if the needle aspirate is non- diagnostic, then the Auris forceps is inserted and 4–6 transbronchial biopsies are performed under fluoroscopy guidance. Specimens are then deployed on slides for touch preps and then transferred into a formalin container for subsequent formalin fixed paraffin embedded (FFPE) tissue analysis. Touch preps are performed for rapid on-site evaluation. It’s relevant to remember that saline on the slides (both for needle aspirations and for the touch preps from tissue biopsy samples) could degrade the cells and make the on-site specimen interpretation difficult or impossible. That is why we strive to avoid instilling saline during RAB.

#### Optimizing the field of view

If the small airway cannot be visualized during the bronchoscope advancement when working at the target, we learned to optimize bronchoscopic visualization once in the distal airways by allowing pressure equilibration between the atmosphere and the target airway. This is simply performed by opening the system to atmosphere by transiently disconnecting the proximal valve (Fig. 1c). If this is unsuccessful in terms of visualizing the airway, then 30–60 mls of air can be insufflated while the scope is “relaxed” (by pushing a specific button on the controller); this allows for the distal aspect of the scope to align with the airway axis while the small airway is splinted open during air insufflation. This way, either the needle or the forceps can be seen existing the scope and can be oriented in the desired direction.

## Discussion

Over the last decade, several guided bronchoscopic technologies have been developed to improve the diagnostic yield for sampling peripheral lung lesions. While these technologies have proven to be feasible and safe, they have several limitations which limit their diagnostic yield, but can potentially be overcome by the robotic bronchoscopic systems. VB has 70% diagnostic yield likely because beyond the 5th generation airway bronchoscopists may get disoriented and miss the small peripheral airway leading to the lesion [7]. R-EBUS also has a reported diagnostic yield of 70%, but recent studies show that this may be much lower than initially reported [6, 10]. A recent multicenter randomized controlled study of r-EBUS via a thin scope (4.2 mm OD) and conventional bronchoscopy with fluoroscopy-guided biopsy showed no difference in overall diagnostic yield (49% with REBUS vs 37% for standard bronchoscopy; *p* = 0.11). The yield is dependent upon lesion size, distance from the hilum, sonographic view obtained (concentric vs eccentric) and nodule morphology (solid, mixed or ground glass) [11]. Multimodal bronchoscopy using EMN and r-EBUS has been used to improve the diagnostic yield but respiratory motion, CT-to-body divergence remain constant challenges with the existing EMN platforms [12]. While higher diagnostic yields have been reported in some smaller series, one large registry study showed a diagnostic rate of 57% with r-EBUS alone, 38.5% with EMN alone, and 47% with combined r-EBUS and EMN [6].

These limitations have led several device manufacturers to pursue the development of robotic bronchoscopic technologies that allow operators to navigate through the small airways, offer EMN guidance to find the target airway (s) leading to the lesion and offer stability during work at the target. The system developed by Auris Health and described herein is the first one to receive FDA clearance but Intuitive Surgical (Sunnyvale, CA) may likely launch their platform in 2019. In a cadaveric study, the Auris bronchoscope reached farther into peripheral airways when compared with a conventional thin bronchoscope (by average generation count: 9 vs 6) [9]. This important finding suggests that the improved access to more peripheral airways may be attributed to the improved structural support, rather than a small diameter, provided by the outer sheath (Fig. 1a) which can be locked in a segmental or subsegmental airway before advancing the inner bronchoscope. Additionally, improved ability to make subtle turns due to 4-way steering thereby achieving precise control in accessing smaller airways is an important technological enhancement. I found that in the majority of cases performed to date, RAB allows direct visualization of peripheral airways and of the biopsy tools as they are advanced outside the scope, thus allowing the bronchoscopist to better steer the tools towards the target (Fig. 6).

The exact location of the target is indicated by the robotic system display, but we routinely confirm it by using r-EBUS (Figs. 4 and 5). The relationship between the r-EBUS and the lesion can be precisely deducted as the exact point of contact between the probe and the lesion is often directly visualized (Fig. 4). Once this is done, the inner scope can be locked in position the r-EBUS probe is removed and the instruments advanced through the working channel. I start with 3–4 needle aspirations and preparation of Diff-Quik smears, followed by 4–6 forceps biopsy from which touch preps are being prepared on site and the actual tissue pieces are sent in formalin for FFPE tissue processing and subsequent immunohistochemistry and molecular studies [13]. At the U of C, however, we routinely perform broad molecular profiling on Diff Quik smears, thus even when the diagnosis is made on the first needle pass, we typically perform another 2–3 aspirations to assure adequate material for next generation sequencing testing (13). We learned that optimal specimen handling and processing in the bronchoscopy laboratory and communication with the cytopathology team on site are essential for assuring quality specimen diagnosis. For instance, injecting saline to flush the working channel or splint open the peripheral airway will likely lead to presence of saline on the slide which could compromise interpretation. When the airway view is lost due to small airway diameter, we proceed with one or more of the following: 1) open the proximal valve and inject 30–60 mls of air; 2) “relax” the scope which allows alignment with the airway axis; or 3) retract the scope a few millimeters and re-orient its tip in the airway lumen.

I have not yet experienced any robotic bronchoscopy- related complications (i.e. pneumothorax, pneumomediastinum, hypoxemia, or airway bleeding requiring cold saline or balloon tamponade). In a few cases, however, the navigation guidance [the yellow and green lines on virtual bronchoscopy images (Fig. 3)] had “lagged behind” the actual robotic bronchoscopic navigation (i.e. advancement of the scope). This could make the bronchoscopist “being lost” in the periphery of the lung. I have learned to troubleshoot this issue by slowing down the scope advancement and by doing my best to keep the scope co-axial (in the center of the airway). In these regards, I found it extremely valuable to create my own map (aka “mental planning”) based on my own understanding of nodule location and airways leading to it. I suspect we will learn more about safety of RAB and potential mechanical or software issues once original studies get published in peer reviewed journals. The health -economic analysis at this point is not possible without analyzing the cost with respect to outcomes. While a cost-effectiveness of any new technology is important, papers focused on health-economics and, in particular, on calculating the cost of the procedures, are based on a large number of robotic procedures performed. This analysis should also consider the outcomes of the procedures, such as a diagnostic yield based on 2-year follow-up. Regarding the procedure charges, our institution codes robotic procedures the same as we code EMN bronchoscopies.

## Conclusions

Based on the initial experience with this novel technology, I believe that the post-marketing robotic-assisted bronchoscopy adoption in community and academic centers will be safe and feasible, with an acceptable and hopefully improved diagnostic yield when compared with the existing technologies. Time will tell if this technology significantly improves yield when compared with the current guided bronchoscopic technologies. One advantage of this robotic system is the stable platform for working in the periphery of the lung for potential bronchoscopic ablative studies. The RAB systems may thus allow the use of microwave ablation, photodynamic therapy or other ablative techniques for inoperable patients with limited stage lung cancer or for the treatment of oligometastatic disease.

## Abbreviations

CT: Computed tomography
EM: Electromagnetic
EMN: Electromagnetic navigation
ETT: Endotracheal tube
OD: Outer diameter
PEEP: Positive end-expiratory pressure
RAB: Robotic-assisted bronchoscopy
r-EBUS: Radial endobronchial ultrasound
U of C: University of Chicago
VB: Virtual bronchoscopy
